# Self-reported symptom severity, general health, and impairment in post-acute phases of COVID-19: retrospective cohort study of Swedish public employees

**DOI:** 10.1038/s41598-022-24307-1

**Published:** 2022-11-17

**Authors:** Simon B. Larsson, Gustaf Stukát von Feilitzen, Maria E. Andersson, Per Sikora, Magnus Lindh, Rickard Nordén, Staffan Nilsson, Robert Sigström

**Affiliations:** 1 Department of Infectious Diseases, Institute of Biomedicine at the Sahlgrenska Academy, University of Gothenburg, Gothenburg, Sweden; 2 Department of Addiction and Dependency, Sahlgrenska University Hospital, Region Västra Götaland, Gothenburg, Sweden; 3 Department of Clinical Microbiology, Sahlgrenska University Hospital, Region Västra Götaland, Gothenburg, Sweden; 4 Core Facilities at the Sahlgrenska Academy, University of Gothenburg, Gothenburg, Sweden; 5 Clinical Genomics Gothenburg, Science for Life Laboratories, Gothenburg, Sweden; 6 Department of Laboratory Medicine, Institute of Biomedicine at the Sahlgrenska Academy, University of Gothenburg, Gothenburg, Sweden; 7 Department of Mathematical Sciences, Chalmers University of Technology, Gothenburg, Sweden; 8 Department of Cognition and Old Age Psychiatry, Sahlgrenska University Hospital, Region Västra Götaland, Gothenburg, Sweden; 9 Department of Psychiatry and Neurochemistry, Institute of Neuroscience and Physiology at the Sahlgrenska Academy, University of Gothenburg, Gothenburg, Sweden

**Keywords:** Epidemiology, SARS-CoV-2, Signs and symptoms

## Abstract

This study aimed to examine current symptom severity and general health in a sample of primarily non-hospitalized persons with polymerase chain reaction (PCR) confirmed COVID-19 in comparison to PCR negative controls. During the first quarter of 2021, we conducted an online survey among public employees in West Sweden, with a valid COVID-19 test result. The survey assessed past-month severity of 28 symptoms and signs, self-rated health, the WHO Disability Assessment Schedule (WHODAS) 2.0 and illness severity at the time of test. We linked participants’ responses to their SARS-CoV-2 PCR tests results. We compared COVID-19 positive and negative participants using univariable and multivariable regression analyses. Out of 56,221 invited, 14,222 (25.3%) responded, with a response rate of 50% among SARS-CoV-2 positive individuals. Analysis included 10,194 participants (86.4% women, mean age 45 years) who tested positive 4–12 weeks (N = 1425; subacute) and > 12 weeks (N = 1584; postcovid) prior to the survey, and 7185 PCR negative participants who did not believe that they had had COVID-19. Symptoms were highly prevalent in all groups, with worst symptoms in subacute phase participants, followed by postcovid phase and PCR negative participants. The most specific symptom for COVID-19 was loss of smell or taste. Both WHODAS 2.0 score and self-rated health were worst in subacute participants, and modestly worse in postcovid participants than in negative controls. Female gender, older age and acute illness severity had larger effects on self-rated health and WHODAS 2.0 score in PCR positive participants than in PCR negative. Studies with longer follow-up are needed to determine the long-term improvement after COVID-19.

## Introduction

Individuals infected by the Severe acute respiratory syndrome coronavirus 2 (SARS-CoV-2) may experience persistent or new symptoms after an acute episode of Coronavirus disease 2019 (COVID-19), which patients named long covid already during spring 2020^1^. The World Health Organization (WHO) has arrived at a preliminary consensus clinical case definition of the post COVID-19 condition (hereafter referred to as postcovid)^2^. Postcovid is described as a persistent condition with functionally impairing symptoms, typically present three months or more from the onset of COVID-19, without alternative explanation. Research criteria for postcovid are lacking, limiting the possibility to estimate its incidence among those infected with COVID-19. Numerous studies have identified a high prevalence of symptoms such as fatigue, shortness of breath and cognitive dysfunction in online patient groups^3–5^ or in follow-up studies of COVID-19 patients^6–8^. However, these symptoms are among the most frequently occurring symptoms in the general population^9,10^. Therefore, in order to establish valid diagnostic criteria, and to estimate contribution to disease and impairment, it is important to examine how the health of persons in the post-acute phase of COVID-19 differ from comparable uninfected individuals^11,12,^. A recent prospective study of the dynamics of 23 somatic symptoms following confirmed COVID-19 estimated an excess prevalence of 12.7% of core postcovid symptoms (e.g. anosmia and difficulties breathing) compared to negative controls while other symptoms (e.g. headache) were equally prevalent in negative controls^13^. Additionally, several studies utilizing large health care databases have shown that non-hospitalized COVID-19 patients have an excess burden of several health conditions compared to matched controls with or without other acute viral illnesses^14–16^. However, the total burden of sequelae may not differ from other hospitalized patients^17^. It is clear that while symptom burden^18,19^ and health care consumption^20^ are dramatically increased during the acute and subacute phase (< 12 weeks post infection) of COVID-19 compared to negative controls, they decrease dramatically before the start of the postcovid phase. Long-term health problems after COVID-19 seem to be more common in women, with increasing age, in hospitalized patients and among those with pre-existing medical conditions^16,19,20^. Otherwise, few risk factors have emerged.

Most studies have focused on symptoms and specific medical conditions as sequelae of COVID-19, whereas less is known about functional impairment. One study found that the by far strongest predictor of long-term sick leave (≥ 12 weeks) following COVID-19 was hospitalization^21^. Among non-hospitalized patients, the strongest risk factor for long term sick leave was a history of sick leave during the year preceding the start of the COVID-19 pandemic. Another study found no or small effects of COVID-19 on sick leave ≥ 12 weeks after infection in non-hospitalized patients^22^. In addition to the lack of strict definition of postcovid, studies vary in how the diagnosis was made, from self-report, antibody status or PCR test and very few include a control group.

With the aim to examine the long-term excess in symptom severity and functional impairment following COVID-19, we designed a study where public employees included in an extensive PCR testing program, irrespective of SARS-CoV-2 positivity, were invited to complete an online survey with questions on current symptoms, general health and functional impairment.

## Methods

### Study population

The study followed the Strengthening the Reporting of Observational Studies in Epidemiology (STROBE) reporting guideline (Additional file 1).

Starting in March 2020, Region Västra Götaland, municipalities within the region, and private employers with public funding, offered their employees PCR (Polymerase chain reaction) testing for presence of SARS-CoV-2 in case of symptoms suggestive of COVID-19. The initial rationale was to avoid unnecessary sickness absence among essential workers. Throughout the period, the decision to order a test was on the nearest manager of the employee. Initially, the indication for testing were mild symptoms (not interfering with ability to work) compatible with COVID-19 with a duration of 24 h. As availability of tests increased and recommendations on contact tracing changed, this decision was increasingly done also in asymptomatic persons.

**Figure 1:**
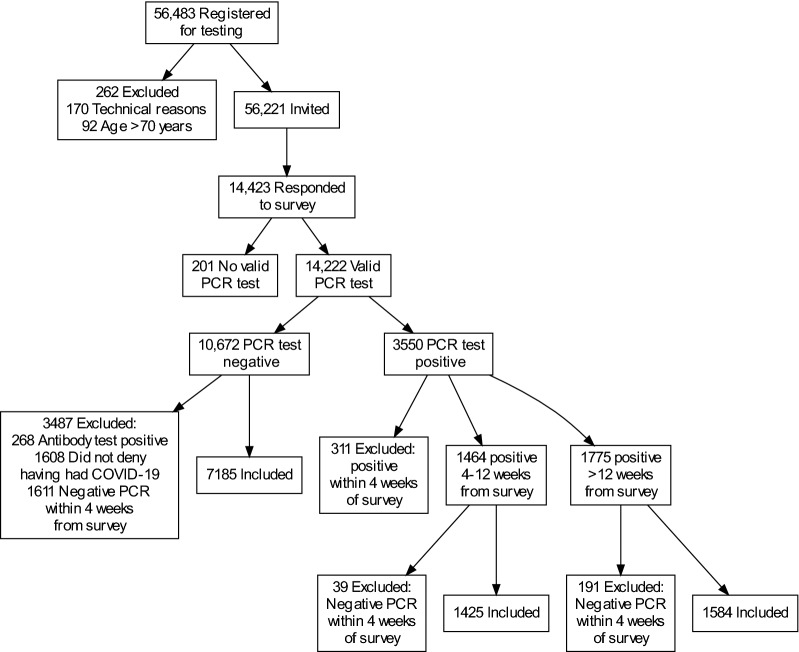
Flowchart of the study sample.

From a database at the Sahlgrenska University Hospital, we identified 56,483 individuals > 18 years old who had registered their Swedish personal identification number, mobile phone number and home address to receive at least one employer-ordered test for presence of SARS-CoV-2 by PCR between March 26th and November 30th, 2020. We excluded individuals whom we were not able to contact for technical reasons (N = 170) and individuals > 70 (N = 92) years of age, as they were unlikely to be employees, leaving 56,221 individuals eligible for the study. A flowchart of the study population is presented in Fig. 1.

All eligible individuals were invited by SMS with a link to the study’s website that contained a comprehensive study description and conditions of participation. To give digital informed consent, participants accepted by accessing the questionnaire using their Swedish electronic identification, Bank-ID, answering all questions and submitting their answer. We used the built-in questionnaire platform of Swedish Healthcare Guide online, 1177.se, Sweden's national healthcare hub, operated by Inera AB on behalf of the regions of Sweden. We could only view and export data from participants who had completed and submitted the questionnaire in its whole. Due to ethical and privacy reasons, we could not ascertain how many persons who started the survey without completing it. With about a week’s interval, two reminders were sent out to individuals who had not yet responded to the survey.

At study begin, we took measures to raise awareness of the study. A brief notice was put on the intranet of Region Västra Götaland and distributed via a COVID-19 newsletter to municipalities. A press release was distributed to local media outlets, resulting in coverage in local radio channels, television news and newspapers.

### Inclusion and exclusion criteria for the present study

As the present study focused on long-term general health and symptoms of those with PCR verified SARS-CoV-2 infection in relation to comparable controls, we applied the following exclusion criteria (Fig. 1): (i) no valid PCR test in the hospital database, (ii) positive test for SARS-CoV-2 within 4 weeks of completing the survey, (iii) negative PCR test but positive serology for SARS-CoV-2, (iv) in people with negative PCR tests and negative or absent serology: not denying having had COVID-19 (to minimize the possibility that participants with an undocumented infection were included in the negative comparison group) and (v) negative test within 4 weeks of completing the survey (to minimize the possibility that current health and symptoms was affected by other acute illness).

We divided our sample into three groups: those in the subacute phase after testing positive (> 4 to ≤ 12 weeks from the survey, hereafter referred to as ‘subacute’) and those in the postcovid phase after testing positive (> 12 weeks from the survey, hereafter referred to as ‘postcovid’) and those who tested negative for SARS-CoV-2 with PCR (hereafter referred to as ‘PCR negative’).

### Data sources

Age and sex were derived from the participant’s Swedish personal identification number. Participants’ place of living was broadly categorized by use of Swedish postal code areas.

### Online survey

#### Participant characteristics

The online survey consisted of about 70 questions and took a median (IQR) of 11 (8–15) minutes to complete. Self-reported occupation was categorized by following the Swedish version of the International Standard Classification of Occupations (ISCO-08) as closely as possible^23^. To capture chronic and pre-pandemic medical conditions, participants were asked if they currently and for at least one year had received treatment or supervision for a number of medical conditions: hypertension, other cardiovascular disease, diabetes mellitus type 1 and type 2, mental disorder, asthma, allergy, thyroid illness and other autoimmune disease. We calculated BMI from self-reported weight and length. BMI could not be calculated for five participants due to obviously unlikely reported values. We also asked to what extent the participants had been exposed to COVID-19 patients at work.

#### General health and functional impairment

We administered the Swedish translation of the self-administered 12-item version of the WHO Disability Assessment Schedule (WHODAS) 2.0 to assess functional impairment^24^, previously found to have good psychometric properties when administered online^25^. Each item is rated on a five-point scale ranging from 0 (No problem) to 4 (Extreme/cannot do). We used the simple scoring method, yielding a range of possible scores 0–48, which has a very high correlation to a more complex scoring method^26^. We also asked a single question about participants’ self-rated health as formulated by the World Health Organization, translated into Swedish by the Public Health Agency of Sweden^27,28^. This question had five alternative responses (very good–very poor). Self-rated health and WHODAS 2.0 sum score had a moderate-strong correlation (Spearman’s ρ = 0.55, p < 0.001). Lastly, we asked if the participant believed that they had had COVID-19.

#### Symptom severity

We asked about presence of 28 different symptoms and signs during the last 30 days. These were selected in October 2020 after review of then available studies of predominantly non-hospitalized patients with persistent symptoms following confirmed or probable COVID-19^29–32^. Participants reported each symptom on a four-point scale (no, mild, moderate or severe complaints) in the same fashion as in previously used symptom scales^10^. Loss of taste (loss of ability to taste salt, sweet, sour and bitter) and loss of smell were asked for separately and could be reported as being not, partly or completely present. As responses to these two questions were strongly correlated (Spearman’s ρ = 0.81, p < 0.001) and it could be questioned whether participants were able to distinguish anosmia from ageusia, we collapsed answers into a single variable containing for each participant their highest rating for anosmia or ageusia. Experience of fever and increased resting heart rate could be reported as not present, subjective presence not verified by measurement, or presence verified by measurement (temperature > 37.9 °C or resting heart rate > 100 beats per minute). Among participants included in the present study (N = 10,194), the symptom survey had a high internal consistency (Cronbach’s α = 0.89).

We further asked whether, and at what level (primary care, specialized outpatient clinic, emergency department, hospitalization), patients had sought care for their complaints within the last 30 days.

#### Questions regarding symptoms at testing

We asked about the severity of illness at the time of testing. Participants were asked to refer to the first time they tested positive, if they had ever done so. If they had never tested positive, we arbitrarily asked them to refer to their first negative test.

### Establishment of past SARS-CoV-2 infection

#### Reverse transcription real-time polymerase chain reaction

Samples for detection of SARS-CoV-2 RNA were collected through two different techniques. Individuals working in hospitals were sampled by experienced personnel through nasopharyngeal and pharyngeal swabs. In other settings, employees used a kit where they sampled their own nostril, pharynx, and saliva.

Most samples were analysed with reverse transcription real-time PCR using the Cobas(R) 6800 kit (Roche Diagnostics, Rotkreuz, Switzerland) for SARS-CoV-2. Due to the high number of samples arriving to the laboratory, additional platforms were also used for detection of SARS-CoV-2 RNA; an in-house one-step real-time-PCR^33^ and two external labs (SciLife Lab, Stockholm, Sweden and EuroFins, Germany).

We retrieved all participants’ PCR results from the laboratory database at the Sahlgrenska University Hospital, including all employer-ordered PCR tests of the invited participants.

### Statistical analysis

Our main interest was to examine general health and symptom severity in postcovid participants, compared to PCR-negative participants. To examine the dynamics of symptoms and general health following COVID-19 we also wanted to compare postcovid participants to subacute participants. First, we compared the postcovid, subacute and negative groups using univariable and multivariable regression analyses, with PCR-negative participants as reference group. We adjusted for exposure to COVID-19 patients at work and occupation, since these two variables could act as confounders. We used negative binomial regression to analyze WHODAS 2.0 scores, due to their skewed, discrete distribution. Results are presented as mean ratios (MR:s) with an MR > 1 indicating a higher mean WHODAS 2.0 score. Self-rated health was analyzed using proportional odds ordinal logistic regression. Results are presented as odds ratios (OR:s) with an OR > 1 indicating a more severe rating of self-rated health. Next, we explored if determinants of WHODAS 2.0 score and self-rated health differed depending on COVID-19 status. We conducted models including each variable of interest, COVID-19 status, and their interaction term. If the interaction term was significant after application of a false discovery rate (FDR) of 10%^34^, we conducted stratified analyses. Further, we calculated absolute proportions with each symptom, by severity, in each group and estimated the odds for more severe symptoms in each of the positive groups using an univariable proportional odds ordinal logistic regression analysis.

Finally, we examined the association between time since positive PCR and outcomes within each of the subacute and postcovid groups. We conducted the same regression analyses as described above, but within each group of positive cases (subacute and postcovid), and with time since positive test as the main variable of interest. Estimates were adjusted for factors that we thought could be related to earlier exposure to the virus: acute illness severity, exposure to COVID-19 patients at work, and occupation. Unless otherwise stated, the significance level was set at at a two-tailed p-value 0.05. We used SPSS v. 27 (IBM Corp., Armonk, NY, USA) for data management, Stata v. 17 (Stata corp., College Station, TX, USA) for data analyses and the R package ggplot2 (v. 3.3.3)^35^ for graphics.

### Ethics approval and consent to participate

The study was approved by the Swedish Ethical Review Authority (reference number 2020-05752) and conducted according to the Helsinki declaration. All participants gave digital informed consent.

## Results

### Recruitment and drop-out analysis

In total, 56,221 individuals were invited to the study, of which 14,222 completed the online survey between January 26 and March 5, 2021, and had a valid PCR test in the laboratory database (response rate 25.3%, Fig. 1). Compared to all those (N = 41,999) not included in the study sample, participants were more likely to be female (86.4% vs. 79.3%, p < 0.001) and were older (mean 45, SD 12 years vs. mean 42, SD 13 years, p < 0.001). Based on aggregated data on test positivity among all invited participants, we estimate a higher response rate among those who had tested positive vs. negative at completion of the survey (50% vs. 20%).

Among the 14,222 participants, 3550 (25.0%) were PCR positive. After excluding participants meeting exclusion criteria for the present study (Fig. 1), we analyzed data from 1425 subacute participants, 1584 postcovid participants and 7185 PCR negative participants.

### Characteristics of participants in the present study

Table 1 presents characteristics of the subacute, postcovid and PCR negative groups. The median age was 45 years and about 85% were women. Weeks (IQR) between test and survey were a median of 8.4 (6.4–10.1) for subacute, 31.8 (14.4–37.4) for postcovid and 23.0 (15.7–33.7) for PCR negative participants. The most marked differences between positive and negative groups were in the proportion who had worked with COVID-19 patients (more common in the positive groups), occupation group (a higher proportion of non-health professionals in the negative group) and in acute illness severity; the majority of those who had tested positive for COVID-19 were bedridden during the acute phase, while most negative participants were without or with mild symptoms at the time of testing. Less than 5% in each group had sought care or been hospitalized in adjunction to testing.

**Table 1:** Characteristics of the sample according to SARS-CoV-2 PCR status.

	SARS-CoV-2 PCR status			p-value
	Subacute positive	Postcovid positive	Negative	
	N = 1425	N = 1584	N = 7185	
Weeks between PCR and survey*, median (IQR)	8.4 (6.4–10.1)	31.8 (14.4–37.4)	23.0 (15.7–33.7)	N/A
Number of tests, median (IQR)	3.0 (2.0–4.0)	2.0 (1.0–2.0)	2.0 (1.0–3.0)	< 0.001^†^
Age, mean (SD)	44.4 (11.8)	44.6 (12.4)	45.0 (12.1)	0.14^‡^
Female	1248 (87.6%)	1320 (83.3%)	6181 (86.0%)	0.003
**Occupation**				< 0.001
Manager	62 (4.4%)	86 (5.4%)	461 (6.4%)	
Health professional	375 (26.3%)	535 (33.8%)	1906 (26.5%)	
Other/associate professional	242 (17.0%)	222 (14.0%)	1774 (24.7%)	
Care worker	678 (47.6%)	650 (41.0%)	2502 (34.8%)	
Other occupation/unknown	68 (4.8%)	91 (5.7%)	542 (7.5%)	
**Place of living**				< 0.001
Gothenburg urban area	805 (56.5%)	991 (62.6%)	3830 (53.3%)	
Other urban area	389 (27.3%)	362 (22.9%)	2142 (29.8%)	
Rural	228 (16.0%)	213 (13.4%)	1139 (15.9%)	
Other part of Sweden/unknown	3 (0.2%)	18 (1.1%)	74 (1.0%)	
**Exposure to COVID-19 patients at work**				< 0.001
No/No patient contact	585 (41.1%)	671 (42.4%)	4567 (63.6%)	
Occasionally	329 (23.1%)	341 (21.5%)	1412 (19.7%)	
Several times	361 (25.3%)	383 (24.2%)	808 (11.2%)	
Worked exclusively with COVID-19 patients for some period	150 (10.5%)	189 (11.9%)	398 (5.5%)	
BMI, mean (SD)	26.8 (5.1)	26.4 (5.2)	26.3 (5.1)**	0.006^‡^
**Nicotine use**				
No	1091 (76.6%)	1245 (78.6%)	5442 (75.7%)	
Smokeless tobacco	178 (12.5%)	206 (13.0%)	825 (11.5%)	
Smoker	112 (7.9%)	97 (6.1%)	709 (9.9%)	
Tobacco free nicotine product	44 (3.1%)	36 (2.3%)	209 (2.9%)	
**Pre-existing medical conditions**				
Hypertension	151 (10.6%)	167 (10.5%)	810 (11.3%)	0.58
Other cardiovascular disease	30 (2.1%)	35 (2.2%)	176 (2.4%)	0.67
Diabetes mellitus type 1	7 (0.5%)	19 (1.2%)	79 (1.1%)	0.089
Diabetes mellitus type 2	29 (2.0%)	32 (2.0%)	130 (1.8%)	0.76
Asthma	157 (11.0%)	152 (9.6%)	728 (10.1%)	0.43
Allergy	329 (23.1%)	326 (20.6%)	1551 (21.6%)	0.24
Thyroid illness	109 (7.6%)	103 (6.5%)	492 (6.8%)	0.44
Other autoimmune disease	88 (6.2%)	96 (6.1%)	502 (7.0%)	0.27
Mental disorder	225 (15.8%)	181 (11.4%)	1104 (15.4%)	< 0.001
**Illness severity in adjunction to test**				< 0.001
No symptoms	116 (8.1%)	67 (4.2%)	977 (13.6%)	
Mild symptoms	507 (35.6%)	544 (34.3%)	4863 (67.7%)	
Bedridden	753 (52.8%)	904 (57.1%)	1288 (17.9%)	
Needed to seek health care	40 (2.8%)	56 (3.5%)	57 (0.8%)	
Hospitalized	9 (0.6%)	13 (0.8%)	0 (0.0%)	
Vaccinated against COVID-19	233 (16.4%)	375 (23.7%)	1730 (24.1%)	< 0.001
Care seeking for surveyed complaints in the last month***				< 0.001
Have complaints, but no care seeking	691 (48.5%)	723 (45.6%)	2342 (32.6%)	
Primary care center	211 (14.8%)	207 (13.1%)	790 (11.0%)	
Other outpatient (e.g. specialist, physiotherapist)	55 (3.9%)	79 (5.0%)	364 (5.1%)	
Emergency department	36 (2.5%)	21 (1.3%)	66 (0.9%)	
Hospitalized	11 (0.8%)	1 (0.1%)	33 (0.5%)	

Numbers are N (%) unless otherwise indicated.

*IQR* interquartile range, *SD* standard deviation.

P-values for categorical variables are from Pearson's Chi-square tests, and from Kruskall–Wallis tests (^†^) or ANOVA (^‡^) for continuous variables.

*Weeks from first positive test for those ever tested positive. Weeks from first negative test for those never tested positive.

**BMI data was missing for four persons in this group.

***For each participant, only highest level of care is counted.

### Symptom severity

**Figure 2:**
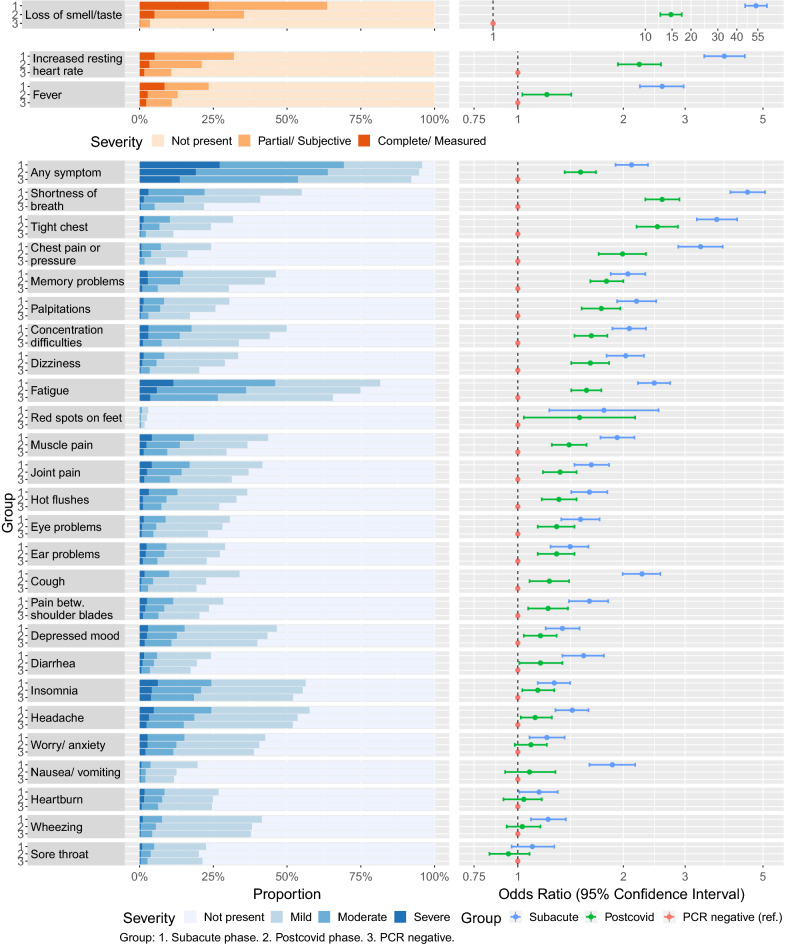
Symptoms and signs as reported by study participants according to subgroup. Left panel: Proportion with each severity level of symptoms and signs among study participants [(1) Subacute phase, (2) postcovid phase, (3) PCR negative]. Right panel: Odds ratio of higher severity of each symptom and sign in SARS-CoV-2 positive in the subacute (blue, > 4 weeks and ≤ 12 weeks from positive PCR) and postcovid (green, > 12 weeks from positive PCR) phase compared to PCR negative participants (red, reference category). Odds ratios are from univariable ordinal logistic regression analyses. The scale is logarithmic.

The prevalence and severity of symptoms is presented in Fig. 2 and Supplementary Tables S1–S2. In all three groups, fatigue was the most common symptom, followed by headache and insomnia, except for shortness of breath as the second most common symptom in the subacute group. All but five symptoms were more severe among those with a positive test > 12 weeks prior to the survey, compared to PCR-negative. Loss of smell or taste had the strongest association to having had COVID-19, followed by shortness of breath.

In the subacute group, 26 out of 28 symptoms were significantly less severe with time since positive test in the multivariable analysis, and for 16 of 28 symptoms, there was a severity reduction of one third or more per 4-week period (Supplementary Table S3). The strongest effect was seen for fever and loss of smell or taste (both OR < 0.40 per four weeks), other respiratory symptoms and fatigue, with a weaker effect of time on neuropsychiatric symptoms.

In the postcovid group, 7 out of 28 symptoms were less severe with time since positive test (Supplementary Table S4). The effect estimates were markedly lower than in the subacute phase, with no symptom showing a higher than 10% reduction in severity odds per 4-week period. The symptoms showing a significant decline in severity with time since positive test were loss of smell or taste, shortness of breath, diarrhoea, palpitations, concentration difficulties, increased resting heart rate and fatigue.

### Functional impairment and self-rated health

**Figure 3:**
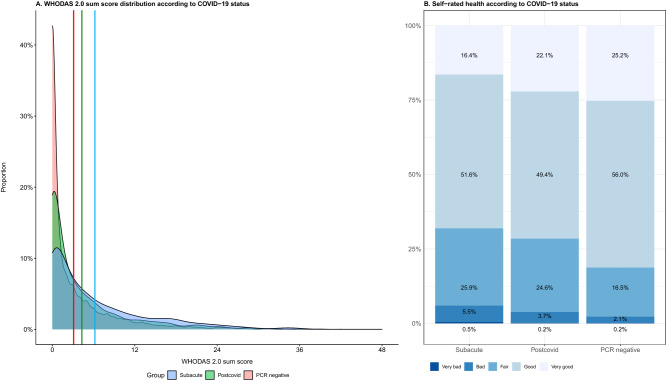
Distribution of WHODAS sum scores according to COVID-19 status. (**A**) Density plot of WHODAS 2.0 sum scores (range 0–48). Vertical axis presents proportion of participants with each WHODAS score (horizontal axis). Vertical lines represent means of PCR negative participants (red; mean 3.1), postcovid (green, mean 4.3) and subacute groups (blue, mean 6.2). (**B**) Proportions in each category of self-rated health, from very bad to very good, left to right: PCR subacute phase, postcovid phase and  negative.

**Figure 4:**
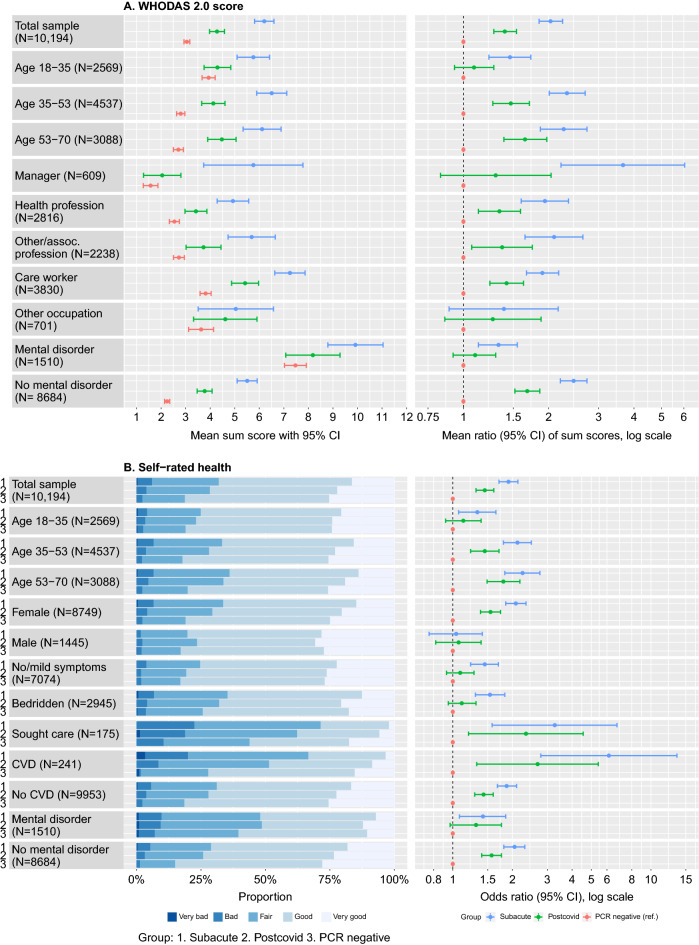
Distribution of WHODAS 2.0 score and rating of self-rated health between subgroups. (**A**) Mean WHODAS 2.0 scores with 95% confidence intervals (left panel) and mean ratio of WHODAS 2.0 scores (right panel, estimated from univariable negative binomial regression analyses) in subgroups where there was a significant interaction between the variable of interest and COVID-19 status on the effect of WHODAS 2.0. (**B**) Proportions of ratings of self-rated health (left panel) and odds ratios for worse self-rated health (right panel, estimated with univariable ordinal logistic regression) in subgroups where there was a significant interaction between the variable of interest and COVID-19 status on the effect of self-rated health.

As can be seen in Fig. 3, postcovid participants had a higher WHODAS 2.0 sum score than PCR-negative participants (mean 4.3 (95% CI 4.0–4.6) vs. 3.1 (95% CI 2.9–3.2), adjusted mean ratio [aMR] 1.40, 95% CI 1.28–1.54, p < 0.001, Fig. 4, Supplementary Table S5), as had subacute participants [mean 6.2 (95% CI 5.8–6.6), aMR 1.97, 95% CI 1.78–2.17, p < 0.001]. Most participants rated their current health as good or very good and a rating of poor or very poor health was relatively uncommon, irrespective of COVID-19 status (Fig. 3, Supplementary Table S6). Adjusted odds ratios for worse self-rated health were in line with the effects on WHODAS 2.0 (Fig. 4, Supplementary Table S6).

To examine if the association between COVID-19 status and WHODAS 2.0 or self-rated health was dependent on other factors, we conducted interaction analyses. In total, 18 out of 80 interaction terms were significant after FDR correction (Supplementary Table S7), indicating that the association depended on that factor. Figure 4, Supplementary Tables S5 and S6 presents stratified analyses of all factors for which there was at least one significant interaction term. As compared to PCR-negative, being in the postcovid phase was related to worse functioning and self-rated health in those above the first age tertile, but not in those below, among women but not among men, in those without a pre-existing mental disorder, but not in those with.

In the subacute group, we found an association between time since positive test and better self-rated health (OR per four-week period 0.75, 95% CI 0.63–0.89, p = 0.001) and lower WHODAS 2.0 sum score (MR per four-week period 0.69, 95% CI 0.61–0.78, p < 0.001) (Supplementary Table S8). In the postcovid group, no such associations were found.

## Discussion

We found that having had COVID-19, verified by PCR, was associated with poorer self-rated health and functioning, and more severe symptoms when compared with patients without a history of COVID-19. These associations were clearly strongest in the subacute phase but persisted to a lesser extent into the postcovid phase. Among participants in the subacute phase, time since positive test was associated with better general health and functioning and lower severity of almost all symptoms, indicating a high rate of improvement during this phase. In the postcovid phase, we found no such association for general health and only for a minority of symptoms.

Previous studies of postcovid in primarily non-hospitalized patients show great variation in design^36^. The condition lacks a formal definition and there are no established measures. This makes comparisons between studies difficult^36,37^. Most studies lack control group or vary in their definition of COVID-19 (i.e., PCR-verified, antibody-positive, or self-reported infection). Typically, studies present the percentage of patients with at least one persisting symptom at follow-up (which can be from four weeks to more than six months), with rates ranging from 25 to 75% in patients > 12 weeks post infection^17,38,39^. Rates are also influenced by methodological factors, such as the number of assessed symptoms and the time frame assessed, i.e. day of survey, past week, past month.

In our study, as in previous studies of symptoms in the general population^9,10^, symptoms that have previously been associated with postcovid were very frequent irrespective of COVID-19 status. Thus, the proportion of participants with a given symptom may be a less useful metric to delineate postcovid from other conditions. While almost all symptoms were more common in the postcovid group compared to PCR-negative, some of the most common symptoms had only a modest association to being in the postcovid group. We found that loss of smell or taste had by far the strongest relationship to having had COVID-19, with shortness of breath, other chest symptoms and cognitive symptoms following, which is in line with previous research^18,40,41^.

Previous studies of hospitalized patients^42^ and in a community sample^43^ showed a decline in symptom burden during the first 12 weeks following COVID-19 but no decline thereafter. Others found continuing decline of some symptoms, such as loss of smell and fatigue, even between 6 and 12 months after infection^38^. Our data also indicate stability of most symptoms postcovid, with the exception of symptoms with the strongest relationship to a positive PCR test (loss of smell or taste, shortness of breath), which had a negative association with time since positive test also in the postcovid group. These symptoms may be more directly related to the infection itself while other symptoms are maintained despite recovery from COVID-19. Highly increased risk for health problems in the subacute phase, but a markedly lower or even non-existent excess risk for adverse outcomes during the postcovid phase have also been found for other conditions such as venous thromboembolism^44^.

We found modest differences in self-rated health and functioning between PCR-negative and postcovid participants that could not be attributed to any measured confounders. However, more than 90% rated their health as at least fair in both groups. One study found no difference in self-reported health between persons who were either positive or negative for SARS-CoV-2 antibodies and with distributions of self-rated health comparable to our study^40^. In line with other studies^19,20,22^, we also found that the effect of COVID-19 on general health was clearly stronger in the subacute phase than in the postcovid phase. Also, as in previous studies^20,22^ COVID-19 had a stronger effect on general health and functioning in older age groups and in women. In males and those aged 18–35 years, there was no differences in general health and functioning between those in the postcovid phase and PCR-negative controls. Further, as expected, self-rated health and functioning was worse in the small group that had sought health care, than in those who had not. However, even among individuals who were bedridden in adjunction to testing, but did not seek care, participants in the postcovid phase did not differ compared to PCR-negative controls. It needs to be emphasized that about 55% of PCR positive participants had been bedridden compared to only 18% of PCR negative participants. The association between severity of initial illness and long-term symptoms after COVID-19 infection is well established^15,45^. Our study suggests that this is the case even within the group infected persons who did not seek care during their acute infection. In fact, a large cohort study found that asymptomatic infections are not associated with adverse outcomes^46^. Similar findings were found for mental health outcomes in a large community sample^47^. The finding that long-term symptoms seem to be less severe among vaccinated persons experiencing breakthrough infection compared to unvaccinated persons^48^, further strengthens this view that the severity of SARS-CoV-2 infection is a major determinant of long-term sequelae. In a recent study from the UK, persons vaccinated with two doses had a 40% reduced risk of persistent symptoms > 12 weeks post infection as compared to unvaccinated persons^49^. As for other determinants of general health and functioning, we found a lesser impact of COVID-19 among individuals with a self-reported mental disorder. This may be explained by the fact that this group had the highest base level of functional impairment of all studied subgroups, indicating a possible ceiling effect.

As with symptoms, we found an association between time since positive test, self-rated health, and functional impairment during the subacute phase, indicating improvement in these measures with longer time since infection. However, no such association was found in postcovid phase participants. A recent longitudinal cohort study, found the same lack of decline of symptom severity > 12 weeks after COVID-19 for “core symptoms of COVID-19” (e.g. chest pain and anosmia), and those symptoms were also elevated as compared to controls (without COVID-19)^13^.

Neither our study nor others can disentangle the mechanism behind that postcovid phase participants seem to have a non-remitting, poorer general health and more severe symptoms than PCR negative participants. A study of 104 patients with postacute sequelae of SARS-CoV-2 infection (PASC) could not identify any specific cause of reported symptoms, despite extensive evaluation including physical examination, laboratory tests and questionnaires^50^. Furthermore, we cannot disentangle the effect of the illness in itself and the knowledge about having had COVID-19. As we could not assume that PCR negative participants had in fact not been infected with SARS-CoV-2, we had to restrict our negative control group to those who in addition did not believe they had had COVID-19. This may bias the results as knowledge of potential problems with long term symptoms following COVID-19 were widespread in the population at the time of the survey. This could have resulted in an increased awareness of symptoms in those who knew they had tested positive for SARS-CoV-2. Such mechanisms seem to be in play in other viral disease with well-established long-term complications. Several studies on self-reported quality of life (QoL) in persons with chronic hepatitis C infection have shown that being aware of having a chronic infection is associated with reduced health-related QoL as compared with persons unaware of their diagnosis^51–53^.

Strengths of the current study include the relatively large number of participants, the comprehensive survey, objectively verified infection and the presence of negative controls.

Our study also has several limitations. First, despite several efforts to increase the response rate, only 25% of those invited completed the survey. Although this is on par with or slightly better than similar studies of COVID-19^54,55^ this was disappointing. Motivation to participate appeared to be strongly affected by personal experience of COVID-19. It is also likely that people with longstanding health problems following COVID-19 were more motivated to participate, which could potentially cause overestimation of associations between COVID-19 and current health status. However, we note that the prevalence of symptoms^9,10^ and level of functional impairment^26^ among PCR negative participants was similar to previous estimates from the general population. This indicates that they are comparable to other populations not infected by SARS-CoV-2. Also, as mentioned above, awareness of having had SARS-CoV-2 could impact the ratings of PCR positive participants.

Secondly, several factors limit the generalizability of our study. Our sample consisted mainly of employed women of working age, with a very small fraction reporting hospitalization from COVID-19. As higher age and more severe acute illness seem to result in more long-term consequences of COVID-19, this may underestimate the severity of long-term consequences of COVID-19 at the population level. However, as discussed above, the postcovid syndrome appears to be more common in women, so overrepresentation of women in our study could be a source of overestimation of long-term consequences. Also, all positive cases in the present study occurred in individuals without prior COVID-19 infection or vaccination. As noted above, long-term consequences may be reduced by vaccination, and plausibly also by previous infection. Finally, the study was conducted when infections were caused by SARS-CoV-2 variants similar to the original Wuhan strain. It is unclear if our findings generalize to more recent SARS-CoV-2 variants but a recent study showed a significantly reduced risk of experiencing long COVID with the omicron variant as compared with the delta variant (OR ≤ 0.5)^56^.

## Conclusions

In this study we found a high prevalence of symptoms in both postcovid patients and PCR negative controls. Symptoms were more severe in postcovid participants than in negative controls, but less severe than in subacute participants. General health and functional impairment were moderately worse in the former group and especially among women and older age groups, but showed significant improvement as compared with participants in the subacute phase. Severity of the acute illness was strongly correlated to worse self-rated health in the long term. While we observed that time since positive test was strongly associated to improved health and symptom severity in the subacute phase, little to no effect of time was seen among postcovid participants. Studies with longer follow-up are needed to determine the long-term improvement after COVID-19.

## Supplementary Information

Supplementary Information.

## Data Availability

The datasets used and/or analysed during the current study are available from the corresponding author on reasonable request.

## Abbreviations

COVID-19: Coronavirus Disease 2019
FDR: False discovery rate
IQR: Inter-quartile range
MR: Mean ratio
OR: Odds ratio
PCR: Polymerase chain reaction
QoL: Quality of Life
SARS-CoV-2: Severe acute respiratory syndrome coronavirus 2
SD: Standard deviation
WHO: World Health Organization
WHODAS: WHO Disability Assessment Schedule
