# Author Correction: Self-reported symptom severity, general health, and impairment in post-acute phases of COVID-19: retrospective cohort study of Swedish public employees

**DOI:** 10.1038/s41598-022-26999-x

**Published:** 2023-01-06

**Authors:** Simon B. Larsson, Gustaf Stukát von Feilitzen, Maria E. Andersson, Per Sikora, Magnus Lindh, Rickard Nordén, Staffan Nilsson, Robert Sigström

**Affiliations:** 1 Department of Infectious Diseases, Institute of Biomedicine at the Sahlgrenska Academy, University of Gothenburg, Gothenburg, Sweden; 2 Department of Addiction and Dependency, Sahlgrenska University Hospital, Region Västra Götaland, Gothenburg, Sweden; 3 Department of Clinical Microbiology, Sahlgrenska University Hospital, Region Västra Götaland, Gothenburg, Sweden; 4 Core Facilities at the Sahlgrenska Academy, University of Gothenburg, Gothenburg, Sweden; 5 Clinical Genomics Gothenburg, Science for Life Laboratories, Gothenburg, Sweden; 6 Department of Laboratory Medicine, Institute of Biomedicine at the Sahlgrenska Academy, University of Gothenburg, Gothenburg, Sweden; 7 Department of Mathematical Sciences, Chalmers University of Technology, Gothenburg, Sweden; 8 Department of Cognition and Old Age Psychiatry, Sahlgrenska University Hospital, Region Västra Götaland, Gothenburg, Sweden; 9 Department of Psychiatry and Neurochemistry, Institute of Neuroscience and Physiology at the Sahlgrenska Academy, University of Gothenburg, Gothenburg, Sweden

Correction to: *Scientific Reports* 10.1038/s41598-022-24307-1, published online 17 November 2022

The original version of this Article contained errors in Figure 4 where the upper panels did not match the legend by colour. In the right lower panel, the order of estimates was incorrectly switched. The original Figure 4 and accompanying legend appear below.

**Figure 4:**
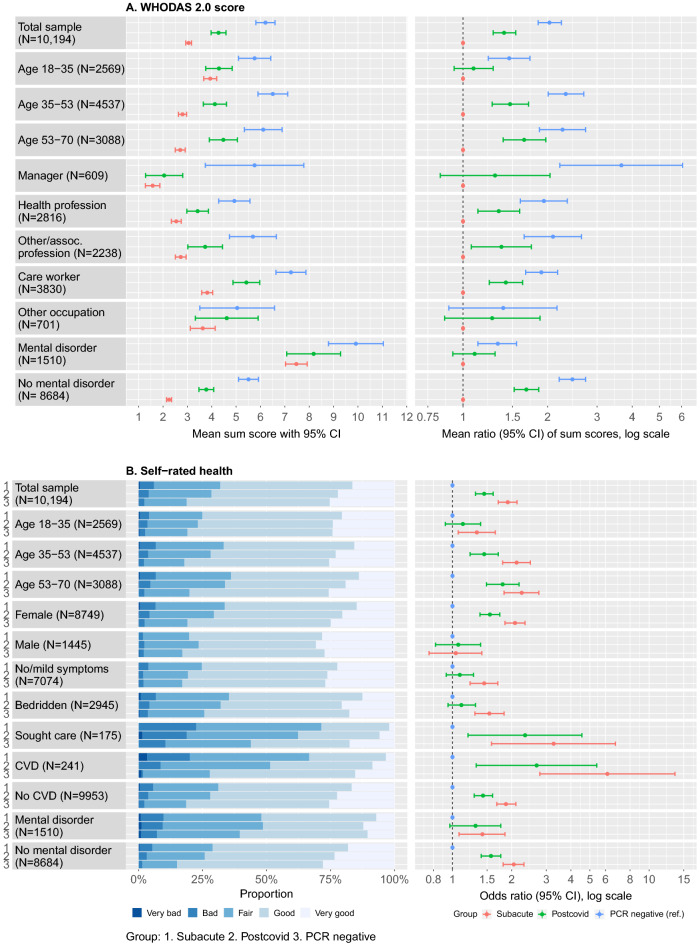
Distribution of WHODAS 2.0 score and rating of self-rated health between subgroups. (**A**) Mean WHODAS 2.0 scores with 95% confidence intervals (left panel) and mean ratio of WHODAS 2.0 scores (right panel, estimated from univariable negative binomial regression analyses) in subgroups where there was a significant interaction between the variable of interest and COVID-19 status on the effect of WHODAS 2.0. (**B**) Proportions of ratings of self-rated health (left panel) and odds ratios for worse self-rated health (right panel, estimated with univariable ordinal logistic regression) in subgroups where there was a significant interaction between the variable of interest and COVID-19 status on the effect of self-rated health.

The original Article has been corrected.

