# Supplementary material for: Self-reported symptom severity, general health, and impairment in post-acute phases of COVID-19: retrospective cohort study of Swedish public employees
Source: Sci Rep. 2022 Nov 17;12:19818. doi: 10.1038/s41598-022-24307-1 (PMC9672032; doi:10.1038/s41598-022-24307-1)
Supplement: Supplementary file 1 — Supplementary Information. [file 41598_2022_24307_MOESM1_ESM.pdf]

Supplementary material to Larsson et al. Self-reported symptom severity, general health, and impairment in post-acute phases of COVID-19: retrospective cohort study of Swedish public employees

Contents:

p. 2	Table S1. Prevalence of symptoms according to severity among subacute phase, postcovid phase and PCR negative participants.
p. 5	Table S2. Relative symptom severity among subacute and postcovid phase COVID-19 positive participants, compared to PCR negative participants
p. 6	Table S3. Effect of time on symptom severity among subacute phase participants
p. 7	Table S4. Effect of time on symptom severity among postcovid phase participants
p. 8	Table S5. Mean scores and relative scores of WHODAS 2.0 in subgroups with significant interaction effects for subacute or postcovid phase participants
p. 9	Table S6. Self-rated health in subgroups with significant interaction effects for subacute or postcovid phase participants
p. 11	Table S7. P-values of the association between interaction terms between COVID-19 status and other variables. and self-rated health and WHODAS 2.0 score
p. 12	Table S8. Association between time since positive test, WHODAS 2.0 score and self-rated health in subacute and postcovid phase participants
p. 13	STROBE statement

Table S1. Prevalence of symptoms according to severity among subacute phase, postcovid phase and PCR negative participants.

	Subacute (N=1425)			Postcovid (N=1584)			Negative (N=7185)		
	N	%	95% CI	N	%	95% CI	N	%	95% CI
**Loss of smell or taste**									
Not present	519	36.4	34.0-38.9	1023	64.6	62.2-66.9	6934	96.5	96.1-96.9
Partial / subjective	572	40.1	37.6-42.7	482	30.4	28.2-32.7	237	3.3	2.9-3.7
Complete / measured	334	23.4	21.3-25.7	79	5.0	4.0-6.1	14	0.2	0.1-0.3
**Increased resting heart rate**									
Not present	970	68.1	65.6-70.5	1251	79.0	76.9-80.9	6415	89.3	88.6-90.0
Partial / subjective	383	26.9	24.6-29.2	281	17.7	15.9-19.7	661	9.2	8.5-9.9
Complete / measured	72	5.1	4.0-6.3	52	3.3	2.5-4.2	109	1.5	1.3-1.8
**Fever**									
Not present	1092	76.6	74.4-78.8	1380	87.1	85.4-88.7	6402	89.1	88.4-89.8
Partial / subjective	213	14.9	13.2-16.9	161	10.2	8.7-11.7	624	8.7	8.1-9.4
Complete / measured	120	8.4	7.1-9.9	43	2.7	2.0-3.6	159	2.2	1.9-2.6
**Any symptom below**									
Not present	60	4.2	3.3-5.3	83	5.2	4.2-6.4	566	7.9	7.3-8.5
Mild	378	26.5	24.3-28.9	490	30.9	28.7-33.2	2762	38.4	37.3-39.6
Moderate	601	42.2	39.6-44.8	709	44.8	42.3-47.2	2879	40.1	38.9-41.2
Severe	386	27.1	24.8-29.4	302	19.1	17.2-21.1	978	13.6	12.8-14.4
**Shortness of breath**									
Not present	642	45.1	42.5-47.6	936	59.1	56.7-61.5	5618	78.2	77.2-79.1
Mild	470	33.0	30.6-35.5	410	25.9	23.8-28.1	1205	16.8	15.9-17.6
Moderate	271	19.0	17.0-21.1	216	13.6	12-15.4	332	4.6	4.2-5.1
Severe	42	2.9	2.2-3.9	22	1.4	0.9-2.1	30	0.4	0.3-0.6
**Tight chest**									
Not present	974	68.4	65.9-70.7	1201	75.8	73.7-77.9	6363	88.6	87.8-89.3
Mild	305	21.4	19.3-23.6	277	17.5	15.7-19.4	673	9.4	8.7-10.1
Moderate	127	8.9	7.5-10.5	95	6.0	4.9-7.2	131	1.8	1.5-2.2
Severe	19	1.3	0.8-2.0	11	0.7	0.4-1.2	18	0.3	0.2-0.4
**Chest pain or pressure**									
Not present	1080	75.8	73.5-78.0	1327	83.8	81.9-85.5	6543	91.1	90.4-91.7
Mild	243	17.1	15.2-19.1	196	12.4	10.8-14.1	526	7.3	6.7-7.9
Moderate	94	6.6	5.4-8.0	50	3.2	2.4-4.1	105	1.5	1.2-1.8
Severe	8	0.6	0.3-1.1	11	0.7	0.4-1.2	11	0.2	0.1-0.3
**Memory problems**									
Not present	767	53.8	51.2-56.4	912	57.6	55.1-60.0	5011	69.7	68.7-70.8
Mild	449	31.5	29.1-34.0	455	28.7	26.5-31.0	1732	24.1	23.1-25.1
Moderate	171	12.0	10.4-13.8	173	10.9	9.5-12.5	381	5.3	4.8-5.8
Severe	38	2.7	1.9-3.6	44	2.8	2.1-3.7	61	0.8	0.7-1.1
**Palpitations**									
Not present	992	69.6	67.2-72.0	1177	74.3	72.1-76.4	5962	83.0	82.1-83.8
Mild	315	22.1	20.0-24.3	297	18.8	16.9-20.7	1012	14.1	13.3-14.9
Moderate	99	6.9	5.7-8.4	95	6.0	4.9-7.2	187	2.6	2.3-3.0
Severe	19	1.3	0.8-2.0	15	0.9	0.6-1.5	24	0.3	0.2-0.5
**Concentration difficulties**									
Not present	715	50.2	47.6-52.8	885	55.9	53.4-58.3	4769	66.4	65.3-67.5
Mild	459	32.2	29.8-34.7	484	30.6	28.3-32.9	1879	26.2	25.1-27.2
Moderate	209	14.7	12.9-16.6	169	10.7	9.2-12.3	458	6.4	5.8-7.0
Severe	42	2.9	2.2-3.9	46	2.9	2.2-3.8	79	1.1	0.9-1.4
**Fatigue**									
Not present	263	18.5	16.5-20.5	398	25.1	23.0-27.3	2477	34.5	33.4-35.6

Mild	507	35.6	33.1-38.1	614	38.8	36.4-41.2	2803	39.0	37.9-40.1
Moderate	492	34.5	32.1-37.0	480	30.3	28.1-32.6	1646	22.9	21.9-23.9
Severe	163	11.4	9.9-13.2	92	5.8	4.7-7.0	259	3.6	3.2-4.1
**Red spots on feet**									
Not present	1384	97.1	96.2-97.9	1545	97.5	96.7-98.2	7066	98.3	98.0-98.6
Mild	30	2.1	1.5-3.0	32	2.0	1.4-2.8	85	1.2	1.0-1.5
Moderate	11	0.8	0.4-1.3	6	0.4	0.2-0.8	29	0.4	0.3-0.6
Severe	0	0	0.0-0.0	1	0.1	0-0.3	5	0.1	0-0.2
**Muscle pain**									
Not present	804	56.4	53.8-59.0	1005	63.4	61.1-65.8	5067	70.5	69.5-71.6
Mild	359	25.2	23.0-27.5	363	22.9	20.9-25.0	1445	20.1	19.2-21.1
Moderate	203	14.2	12.5-16.1	180	11.4	9.9-13.0	578	8.0	7.4-8.7
Severe	59	4.1	3.2-5.3	36	2.3	1.6-3.1	95	1.3	1.1-1.6
**Joint pain**									
Not present	832	58.4	55.8-60.9	999	63.1	60.7-65.4	4942	68.8	67.7-69.8
Mild	352	24.7	22.5-27.0	360	22.7	20.7-24.8	1509	21.0	20.1-22.0
Moderate	183	12.8	11.2-14.7	185	11.7	10.2-13.3	624	8.7	8.1-9.4
Severe	58	4.1	3.1-5.2	40	2.5	1.8-3.4	110	1.5	1.3-1.8
**Hot flushes**									
Not present	905	63.5	61.0-66.0	1064	67.2	64.8-69.5	5245	73.0	72.0-74.0
Mild	338	23.7	21.6-26.0	376	23.7	21.7-25.9	1411	19.6	18.7-20.6
Moderate	138	9.7	8.2-11.3	126	8.0	6.7-9.4	451	6.3	5.7-6.9
Severe	44	3.1	2.3-4.1	18	1.1	0.7-1.8	78	1.1	0.9-1.3
**Eye problems**									
Not present	989	69.4	67.0-71.8	1140	72.0	69.7-74.1	5523	76.9	75.9-77.8
Mild	311	21.8	19.7-24.0	355	22.4	20.4-24.5	1328	18.5	17.6-19.4
Moderate	105	7.4	6.1-8.8	76	4.8	3.8-5.9	287	4.0	3.6-4.5
Severe	20	1.4	0.9-2.1	13	0.8	0.5-1.4	47	0.7	0.5-0.9
**Ear problems**									
Not present	1012	71.0	68.6-73.3	1153	72.8	70.6-74.9	5552	77.3	76.3-78.2
Mild	284	19.9	17.9-22.1	299	18.9	17.0-20.9	1201	16.7	15.9-17.6
Moderate	96	6.7	5.5-8.1	100	6.3	5.2-7.6	357	5.0	4.5-5.5
Severe	33	2.3	1.6-3.2	32	2.0	1.4-2.8	75	1.0	0.8-1.3
**Cough**									
Not present	942	66.1	63.6-68.5	1227	77.5	75.4-79.5	5799	80.7	79.8-81.6
Mild	341	23.9	21.8-26.2	285	18.0	16.2-19.9	1182	16.5	15.6-17.3
Moderate	119	8.4	7.0-9.9	63	4.0	3.1-5.0	180	2.5	2.2-2.9
Severe	23	1.6	1.1-2.4	9	0.6	0.3-1.0	24	0.3	0.2-0.5
**Pain between shoulder blades**									
Not present	1021	71.6	69.3-73.9	1212	76.5	74.4-78.6	5729	79.7	78.8-80.7
Mild	242	17.0	15.1-19.0	240	15.2	13.5-17.0	1001	13.9	13.1-14.7
Moderate	127	8.9	7.5-10.5	102	6.4	5.3-7.7	378	5.3	4.8-5.8
Severe	35	2.5	1.7-3.4	30	1.9	1.3-2.7	77	1.1	0.9-1.3
**Depressed mood**									
Not present	762	53.5	50.9-56.1	898	56.7	54.2-59.1	4316	60.1	58.9-61.2
Mild	446	31.3	28.9-33.7	486	30.7	28.4-33.0	2093	29.1	28.1-30.2
Moderate	177	12.4	10.8-14.2	161	10.2	8.7-11.7	654	9.1	8.5-9.8
Severe	40	2.8	2.0-3.8	39	2.5	1.8-3.3	122	1.7	1.4-2.0
**Diarrhea**									
Not present	1081	75.9	73.6-78.0	1277	80.6	78.6-82.5	5944	82.7	81.8-83.6
Mild	260	18.2	16.3-20.3	230	14.5	12.9-16.3	992	13.8	13.0-14.6
Moderate	63	4.4	3.4-5.6	62	3.9	3.0-5.0	208	2.9	2.5-3.3
Severe	21	1.5	0.9-2.2	15	0.9	0.6-1.5	41	0.6	0.4-0.8
**Insomnia**									
Not present	623	43.7	41.2-46.3	708	44.7	42.3-47.2	3446	48.0	46.8-49.1
Mild	456	32.0	29.6-34.5	546	34.5	32.2-36.8	2415	33.6	32.5-34.7
Moderate	258	18.1	16.2-20.2	265	16.7	15.0-18.6	1049	14.6	13.8-15.4
Severe	88	6.2	5.0-7.5	65	4.1	3.2-5.2	275	3.8	3.4-4.3

**Headache**									
Not present	604	42.4	39.8-45.0	735	46.4	44.0-48.9	3452	48.0	46.9-49.2
Mild	475	33.3	30.9-35.8	555	35.0	32.7-37.4	2658	37.0	35.9-38.1
Moderate	278	19.5	17.5-21.6	244	15.4	13.7-17.2	909	12.7	11.9-13.4
Severe	68	4.8	3.8-6.0	50	3.2	2.4-4.1	166	2.3	2.0-2.7
Worry / anxiety									
Not present	818	57.4	54.8-60.0	941	59.4	57.0-61.8	4403	61.3	60.1-62.4
Mild	391	27.4	25.2-29.8	446	28.2	26.0-30.4	1963	27.3	26.3-28.4
Moderate	178	12.5	10.9-14.3	155	9.8	8.4-11.3	681	9.5	8.8-10.2
Severe	38	2.7	1.9-3.6	42	2.7	1.9-3.5	138	1.9	1.6-2.3
**Nausea / vomiting**									
Not present	1145	80.4	78.2-82.4	1386	87.5	85.8-89.1	6348	88.4	87.6-89.1
Mild	228	16.0	14.2-18.0	166	10.5	9.0-12.1	700	9.7	9.1-10.4
Moderate	44	3.1	2.3-4.1	27	1.7	1.2-2.4	118	1.6	1.4-2.0
Severe	8	0.6	0.3-1.1	5	0.3	0.1-0.7	19	0.3	0.2-0.4
**Heartburn**									
Not present	1044	73.3	70.9-75.5	1191	75.2	73.0-77.3	5424	75.5	74.5-76.5
Mild	261	18.3	16.4-20.4	272	17.2	15.4-19.1	1312	18.3	17.4-19.2
Moderate	97	6.8	5.6-8.2	98	6.2	5.1-7.5	401	5.6	5.1-6.1
Severe	23	1.6	1.1-2.4	23	1.5	0.9-2.1	48	0.7	0.5-0.9
**Sneezing**									
Not present	835	58.6	56.0-61.1	981	61.9	59.5-64.3	4476	62.3	61.2-63.4
Mild	482	33.8	31.4-36.3	516	32.6	30.3-34.9	2405	33.5	32.4-34.6
Moderate	93	6.5	5.3-7.9	81	5.1	4.1-6.3	284	4.0	3.5-4.4
Severe	15	1.1	0.6-1.7	6	0.4	0.2-0.8	20	0.3	0.2-0.4
**Sore throat**									
Not present	1104	77.5	75.3-79.6	1266	79.9	77.9-81.8	5656	78.7	77.8-79.7
Mild	251	17.6	15.7-19.7	259	16.4	14.6-18.2	1340	18.6	17.8-19.6
Moderate	58	4.1	3.1-5.2	53	3.3	2.5-4.3	168	2.3	2.0-2.7
Severe	12	0.8	0.5-1.4	6	0.4	0.2-0.8	21	0.3	0.2-0.4

Table S2. Relative symptom severity among subacute and postcovid phase COVID-19 positive participants, compared to PCR negative participants

	Subacute			Postcovid		
Symptom	OR	95% CI	P	OR	95% CI	P
Loss of smell or taste	53.53	45.40-63.11	<0.001	14.77	12.57-17.37	<0.001
Shortness of breath	4.52	4.04-5.07	<0.001	2.58	2.31-2.89	<0.001
Increased resting heart rate	3.88	3.40-4.44	<0.001	2.22	1.93-2.56	<0.001
Tight chest	3.69	3.24-4.22	<0.001	2.50	2.18-2.86	<0.001
Chest pain or pressure	3.32	2.87-3.84	<0.001	1.99	1.70-2.32	<0.001
Fever	2.58	2.23-2.97	<0.001	1.21	1.03-1.42	0.024
Fatigue	2.45	2.20-2.72	<0.001	1.57	1.42-1.73	<0.001
Cough	2.26	1.99-2.55	<0.001	1.23	1.08-1.40	0.002
Palpitations	2.18	1.92-2.48	<0.001	1.73	1.52-1.96	<0.001
Any symptom	2.11	1.90-2.35	<0.001	1.51	1.36-1.67	<0.001
Concentration difficulties	2.08	1.86-2.32	<0.001	1.62	1.45-1.80	<0.001
Memory problems	2.06	1.84-2.31	<0.001	1.79	1.61-2.00	<0.001
Dizziness	2.03	1.79-2.29	<0.001	1.61	1.42-1.82	<0.001
Muscle pain	1.92	1.72-2.15	<0.001	1.40	1.25-1.57	<0.001
Nausea/vomiting	1.86	1.60-2.16	<0.001	1.08	0.92-1.28	0.344
Red spots on feet	1.76	1.23-2.52	0.002	1.50	1.04-2.16	0.031
Joint pain	1.62	1.45-1.82	<0.001	1.32	1.18-1.47	<0.001
Pain between shoulderblades	1.60	1.40-1.81	<0.001	1.22	1.07-1.39	0.002
Hot flushes	1.60	1.42-1.80	<0.001	1.31	1.17-1.47	<0.001
Diarrhea	1.54	1.34-1.76	<0.001	1.16	1.01-1.34	0.031
Eye problems	1.51	1.33-1.71	<0.001	1.29	1.14-1.45	<0.001
Headache	1.43	1.28-1.59	<0.001	1.12	1.02-1.25	0.025
Ear problems	1.41	1.24-1.59	<0.001	1.29	1.14-1.45	<0.001
Depressed mood	1.34	1.20-1.50	<0.001	1.16	1.04-1.29	0.007
Insomnia	1.27	1.14-1.41	<0.001	1.14	1.03-1.27	0.009
Sneezing	1.22	1.09-1.37	0.001	1.03	0.93-1.16	0.545
Worry/anxiety	1.21	1.08-1.36	0.001	1.09	0.98-1.21	0.127
Heartburn	1.15	1.01-1.30	0.034	1.04	0.91-1.17	0.581
Sore throat	1.10	0.96-1.27	0.151	0.94	0.83-1.08	0.407

Abbreviations: OR: Odds ratio, estimated from a univariable ordinal logistic regression; 95% CI: 95% Confidence interval

Table S3. Effect of time on symptom severity among subacute phase participants

	Univariable			Multivariable		
	OR per 4 weeks	95% CI	P	OR per 4 weeks	95% CI	P
Fever	0.30	0.23-0.37	**<0.001**	0.28	0.22-0.35	**<0.001**
Loss of taste or smell	0.37	0.31-0.44	**<0.001**	0.36	0.30-0.44	**<0.001**
Cough	0.46	0.38-0.56	**<0.001**	0.44	0.36-0.54	**<0.001**
Sore throat	0.52	0.42-0.65	**<0.001**	0.49	0.39-0.61	**<0.001**
Tight chest	0.56	0.46-0.68	**<0.001**	0.52	0.42-0.64	**<0.001**
Fatigue	0.57	0.48-0.68	**<0.001**	0.53	0.45-0.64	**<0.001**
Muscle pain	0.60	0.50-0.72	**<0.001**	0.58	0.48-0.70	**<0.001**
Headache	0.61	0.51-0.73	**<0.001**	0.60	0.50-0.71	**<0.001**
Chest pain or pressure	0.61	0.49-0.76	**<0.001**	0.56	0.45-0.70	**<0.001**
Sneezing	0.63	0.52-0.76	**<0.001**	0.63	0.52-0.76	**<0.001**
Nausea/vomiting	0.65	0.52-0.82	**<0.001**	0.65	0.51-0.82	**<0.001**
Worry/anxiety	0.66	0.55-0.79	**<0.001**	0.63	0.53-0.76	**<0.001**
Diarrhea	0.66	0.54-0.82	**<0.001**	0.66	0.53-0.82	**<0.001**
Shortness of breath	0.67	0.56-0.80	**<0.001**	0.62	0.52-0.74	**<0.001**
Red spots on feet	0.67	0.39-1.15	0.144	0.70	0.40-1.21	0.201
Increased resting heart rate	0.67	0.55-0.81	**<0.001**	0.65	0.53-0.79	**<0.001**
Depressed mood	0.68	0.57-0.81	**<0.001**	0.65	0.54-0.78	**<0.001**
Joint pain	0.70	0.59-0.84	**<0.001**	0.67	0.56-0.81	**<0.001**
Palpitations	0.70	0.57-0.85	**<0.001**	0.68	0.56-0.83	**<0.001**
Concentration difficulties	0.73	0.61-0.87	**<0.001**	0.69	0.58-0.83	**<0.001**
Dizziness	0.73	0.60-0.88	**0.001**	0.69	0.57-0.84	**<0.001**
Pain between shoulderblades	0.73	0.60-0.89	**0.002**	0.69	0.56-0.85	**0.001**
Heartburn	0.75	0.61-0.91	**0.005**	0.71	0.58-0.88	**0.002**
Insomnia	0.75	0.64-0.89	**0.001**	0.73	0.61-0.87	**<0.001**
Hot flushes	0.78	0.64-0.94	**0.008**	0.76	0.63-0.92	**0.006**
Eye problems	0.82	0.68-1.00	0.054	0.78	0.64-0.96	**0.017**
Memory problems	0.83	0.70-0.99	**0.039**	0.79	0.66-0.94	**0.009**
Ear problems	0.84	0.69-1.03	0.096	0.82	0.67-1.01	0.056

Results are from ordinal logistic regression analyses with time since positive PCR as independent variable. An OR <1 represents less severe symptom with longer time since positive PCR. OR:s are per four-week period
Multivariable model: adjusted for illness severity at time of testing, occupation group and COVID-19 exposure at work

Table S4. Effect of time on symptom severity among postcovid phase participants

	Univariable			Multivariable		
	OR per 4 weeks	95% CI	P	OR per 4 weeks	95% CI	P
Loss of smell or taste	0.93	0.90-0.96	**<0.001**	0.93	0.89-0.96	**<0.001**
Shortness of breath	0.93	0.90-0.97	**<0.001**	0.94	0.90-0.97	**<0.001**
Diarrhea	0.95	0.91-0.99	**0.025**	0.95	0.91-0.99	**0.025**
Palpitations	0.96	0.92-1.00	**0.028**	0.95	0.91-0.99	**0.013**
Red spots on feet	0.96	0.86-1.08	0.500	0.95	0.84-1.07	0.377
Concentration difficulties	0.96	0.93-0.99	**0.018**	0.96	0.93-1.00	**0.026**
Increased resting heart rate	0.97	0.93-1.02	0.209	0.96	0.91-1.00	**0.045**
Chest pain or pressure	0.96	0.92-1.01	0.118	0.97	0.92-1.02	0.246
Fatigue	0.97	0.94-1.00	0.072	0.97	0.93-1.00	**0.043**
Tight chest	0.97	0.93-1.01	0.122	0.97	0.93-1.01	0.191
Memory problems	0.97	0.93-1.00	0.051	0.97	0.94-1.01	0.168
Headache	0.98	0.95-1.01	0.208	0.98	0.95-1.01	0.261
Cough	0.98	0.94-1.02	0.309	0.98	0.94-1.02	0.328
Depressed mood	0.99	0.95-1.02	0.407	0.98	0.95-1.02	0.283
Eye problems	0.99	0.96-1.03	0.736	0.98	0.95-1.02	0.439
Fever	0.99	0.94-1.04	0.681	0.98	0.92-1.03	0.399
Insomnia	0.99	0.96-1.02	0.502	0.99	0.96-1.02	0.530
Dizziness	0.99	0.95-1.02	0.466	0.99	0.95-1.03	0.506
Sneezing	0.99	0.96-1.03	0.581	0.99	0.96-1.03	0.698
Muscle pain	1.00	0.97-1.04	0.909	0.99	0.96-1.03	0.761
Worry/anxiety	0.99	0.96-1.03	0.657	1.00	0.96-1.03	0.831
Hot flushes	1.00	0.96-1.04	0.974	1.00	0.96-1.04	0.955
Ear problems	1.00	0.97-1.04	0.827	1.01	0.97-1.05	0.704
Nausea/vomiting	1.01	0.96-1.06	0.721	1.01	0.96-1.07	0.698
Heartburn	1.02	0.98-1.06	0.338	1.02	0.97-1.06	0.443
Joint pain	1.03	0.99-1.06	0.120	1.03	0.99-1.07	0.127
Sore throat	1.03	0.99-1.08	0.188	1.03	0.98-1.08	0.252
Pain between shoulderblades	1.03	0.98-1.07	0.219	1.03	0.99-1.07	0.203

Results are from ordinal logistic regressions with time since positive PCR as independent variable. An OR <1 represents less severe symptom with longer time since positive PCR. OR:s are per four-week period
Multivariable model: adjusted for illness severity at time of testing, occupation group and COVID-19 exposure at work

Table S5. Mean scores and relative scores of WHODAS 2.0 in subgroups with significant interaction effects for subacute or postcovid phase participants

	Subacute			Postcovid			Negative				Subacute				Postcovid		
	n	Mean	95% CI	n	Mean	95% CI	n	Mean	95% CI		MR	95% CI	P		MR	95% CI	P
**Total sample (N=10194)**	1425	6.2	5.8-6.6	1584	4.3	4.0-4.6	7185	3.1	2.9-3.2		2.03	1.85-2.24	<0.001		1.40	1.28-1.54	<0.001
Age 18-35 (N=2569)	365	5.8	5.1-6.4	436	4.3	3.8-4.8	1768	3.9	3.7-4.2		1.46	1.23-1.73	<0.001		1.09	0.93-1.28	0.286
Age 35-53 (N=4537)	656	6.5	5.9-7.1	671	4.1	3.7-4.6	3210	2.8	2.6-3.0		2.32	2.01-2.69	<0.001		1.47	1.27-1.71	<0.001
Age 53-70 (N=3088)	404	6.1	5.3-6.9	477	4.5	3.9-5.1	2207	2.7	2.5-2.9		2.26	1.87-2.73	<0.001		1.65	1.39-1.97	<0.001
Female (N=8749)	1248	6.5	6.1-7.0	1320	4.5	4.2-4.9	6181	3.1	3.0-3.3		2.08	1.88-2.30	<0.001		1.44	1.30-1.59	<0.001
Male (N=1445)	177	3.9	3.0-4.9	264	3	2.5-3.6	1004	2.5	2.2-2.8		1.57	1.18-2.09	0.002		1.21	0.95-1.55	0.122
Occupation																	
Manager (N=609)	62	5.8	3.7-7.8	86	2	1.3-2.8	461	1.6	1.3-1.9		3.66	2.21-6.04	<0.001		1.30	0.83-2.04	0.257
Health professional (N=2816)	375	4.9	4.3-5.6	535	3.4	3.0-3.9	1906	2.5	2.3-2.7		1.94	1.60-2.35	<0.001		1.34	1.13-1.59	0.001
Other/associate professional (N=2238)	242	5.7	4.7-6.7	222	3.7	3.0-4.4	1774	2.7	2.5-2.9		2.09	1.65-2.64	<0.001		1.37	1.07-1.75	0.013
Care worker (N=3830)	678	7.2	6.6-7.9	650	5.4	4.9-6.0	2502	3.8	3.6-4.0		1.90	1.67-2.17	<0.001		1.42	1.24-1.63	<0.001
Other/unknown occupation (N=701)	68	5	3.5-6.6	91	4.6	3.3-5.9	542	3.6	3.1-4.1		1.39	0.89-2.16	0.144		1.27	0.86-1.88	0.227
Mental disorder (N=1510)	225	9.9	8.8-11.0	181	8.2	7.1-9.3	1104	7.5	7.0-7.9		1.33	1.13-1.55	<0.001		1.10	0.92-1.30	0.303
No mental disorder (N=8684)	1200	5.5	5.1-5.9	1403	3.8	3.5-4.1	6081	2.2	2.1-2.4		2.45	2.20-2.73	<0.001		1.68	1.52-1.86	<0.001

Abbreviations: MR: Mean ratio, estimated from a univariable negative binomial regression; 95% CI: 95% Confidence interval

An MR > 1 indicates association with higher WHODAS 2.0 score, using PCR negative participants as reference

Table S6. Self-rated health in subgroups with significant interaction effects for subacute or postcovid phase participants

		Subacute		Postcovid		Negative		Subacute			Postcovid		
		N	%	N	%	N	%	OR	95% CI	P	OR	95% CI	P
**Total sample**	Very bad	7	0.5	3	0.2	15	0.2	1.91	1.71-2.13	<0.001	1.45	1.31-1.61	<0.001
	Bad	79	5.5	59	3.7	149	2.1						
	Fair	369	25.9	389	24.6	1186	16.5						
	Good	736	51.6	783	49.4	4021	56.0						
	Very good	234	16.4	350	22.1	1814	25.2						
	Total	1425	100.0	1584	100.0	7185	100.0						
Age 18-35 (N=2569)	Very bad	2	0.5	1	0.2	7	0.4	1.33	1.07-1.65	0.011	1.13	0.92-1.39	0.238
	Bad	13	3.6	14	3.2	39	2.2						
	Fair	76	20.8	86	19.7	291	16.5						
	Good	199	54.5	230	52.8	1003	56.7						
	Very good	75	20.5	105	24.1	428	24.2						
	Total	365	100.0	436	100.0	1768	100.0						
Age 35-53 (N=4537)	Very bad	3	0.5	1	0.1	6	0.2	2.12	1.80-2.49	<0.001	1.45	1.23-1.71	<0.001
	Bad	41	6.3	24	3.6	62	1.9						
	Fair	174	26.5	164	24.4	506	15.8						
	Good	335	51.1	328	48.9	1817	56.6						
	Very good	103	15.7	154	23.0	819	25.5						
	Total	656	100.0	671	100.0	3210	100.0						
Age 53-70 (N=3088)	Very bad	2	0.5	1	0.2	2	0.1	2.25	1.83-2.75	<0.001	1.80	1.49-2.18	<0.001
	Bad	25	6.2	21	4.4	48	2.2						
	Fair	119	29.5	139	29.1	389	17.6						
	Good	202	50.0	225	47.2	1201	54.4						
	Very good	56	13.9	91	19.1	567	25.7						
	Total	404	100.0	477	100.0	2207	100.0						
Female (N=8749)	Very bad	7	0.6	3	0.2	12	0.2	2.08	1.85-2.34	<0.001	1.55	1.38-1.74	<0.001
	Bad	76	6.1	53	4.0	132	2.1						
	Fair	337	27.0	333	25.2	1034	16.7						
	Good	644	51.6	662	50.2	3463	56.0						
	Very good	184	14.7	269	20.4	1540	24.9						
	Total	1248	100.0	1320	100.0	6181	100.0						
Male (N=1445)	Very bad	0	0.0	0	0.0	3	0.3	1.04	0.76-1.41	0.814	1.07	0.82-1.39	0.627
	Bad	3	1.7	6	2.3	17	1.7						
	Fair	32	18.1	56	21.2	152	15.1						
	Good	92	52.0	121	45.8	558	55.6						
	Very good	50	28.2	81	30.7	274	27.3						
	Total	177	100.0	264	100.0	1004	100.0						
Acute illness severity													
No or mild symptoms (N=7074)	Very bad	1	0.2	1	0.2	10	0.2	1.45	1.23-1.70	<0.001	1.09	0.93-1.28	0.961
	Bad	23	3.7	10	1.6	101	1.7						
	Fair	130	20.9	107	17.5	883	15.1						
	Good	330	53.0	333	54.5	3269	56						
	Very good	139	22.3	160	26.2	1577	27						

	Total	623	100.0		611	100.0		5840	100.0								
Palpable illness (N=2945)	Very bad	6	0.8		1	0.1		5	0.4		1.54	1.30-1.83	<0.001		1.11	0.95-1.31	0.193
	Bad	45	6.0		37	4.1		42	3.3								
	Fair	215	28.6		252	27.9		284	22								
	Good	393	52.2		428	47.3		730	56.7								
	Very good	94	12.5		186	20.6		227	17.6								
	Total	753	100.0		904	100.0		1288	100.0								
Sought health care (N=175)	Very bad	0	0.0		1	1.4		0	0		3.27	1.58-6.74	0.001		2.34	1.20-4.55	0.012
	Bad	11	22.4		12	17.4		6	10.5								
	Fair	24	49.0		30	43.5		19	33.3								
	Good	13	26.5		22	31.9		22	38.6								
	Very good	1	2.0		4	5.8		10	17.5								
	Total	49	100.0		69	100.0		57	100.0								
Mental disorder (N=1510)	Very bad	2	0.9		2	1.1		10	0.9		1.42	1.08-1.85	0.011		1.31	0.97-1.76	0.080
	Bad	20	8.9		15	8.3		69	6.3								
	Fair	86	38.2		71	39.2		358	32.4								
	Good	101	44.9		71	39.2		551	49.9								
	Very good	16	7.1		22	12.2		116	10.5								
	Total	225	100.0		181	100.0		1104	100.0								
No mental disorder (N=8684)	Very bad	5	0.4		1	0.1		5	0.1		2.05	1.81-2.31	<0.001		1.57	1.40-1.76	<0.001
	Bad	59	4.9		44	3.1		80	1.3								
	Fair	283	23.6		318	22.7		828	13.6								
	Good	635	52.9		712	50.7		3470	57.1								
	Very good	218	18.2		328	23.4		1698	27.9								
	Total	1200	100.0		1403	100.0		6081	100.0								
Cardiovascular disease (N=241)	Very bad	1	3.3		0	0.0		2	1.1		6.13	2.78-13.53	<0.001		2.68	1.32-5.42	0.006
	Bad	5	16.7		3	8.6		1	0.6								
	Fair	14	46.7		15	42.9		46	26.1								
	Good	9	30.0		14	40.0		100	56.8								
	Very good	1	3.3		3	8.6		27	15.3								
	Total	30	100.0		35	100.0		176	100.0								
No cardiovascular disease (N=9953)	Very bad	6	0.4		3	0.2		13	0.2		1.87	1.68-2.10	<0.001		1.43	1.29-1.60	<0.001
	Bad	74	5.3		56	3.6		148	2.1								
	Fair	355	25.4		374	24.1		1140	16.3								
	Good	727	52.1		769	49.6		3921	55.9								
	Very good	233	16.7		347	22.4		1787	25.5								
	Total	1395	100.0		1549	100.0		7009	100.0								
Abbreviations: OR: Odds ratio, estimated from a univariable ordinal logistic regression; 95% CI: 95% Confidence interval																	
An OR > 1 indicates association with worse self-rated health, using PCR negative participants as reference																	

Table S7. P-values of the association between interaction terms between COVID-19 status and other variables and self-rated health and WHODAS 2.0 score

	WHODAS 2.0		Self-rated health	
	Subacute phase	Postcovid phase	Subacute phase	Postcovid phase
Age 18-35 (ref.)	Reference	Reference	Reference	Reference
Age 35-53	<0.001*	0.009*	0.001*	0.059
Age 53-70	0.001*	0.001*	<0.001*	0.001*
Male	Reference	Reference	Reference	Reference
Female	0.059	0.184	<0.001*	0.012
BMI < 25 (ref.)	Reference	Reference	Reference	Reference
BMI 25-29	0.538	0.667	0.444	0.306
BMI 30-	0.938	0.677	0.048	0.608
Occupation				
Manager (ref.)	Reference	Reference	Reference	Reference
Health professional	0.010*	0.878	0.25	0.288
Other/associate professional	0.027	0.831	0.205	0.26
Care worker	0.006*	0.682	0.44	0.555
Other/unknown	0.002*	0.937	0.162	0.925
Acute illness severity				
No symptoms	0.102	0.036	<0.001*	0.015
Mild symptoms (ref.)	Reference	Reference	Reference	Reference
Palpable illness	0.77	0.985	0.061	0.331
Sought health care	0.639	0.65	0.004*	0.008*
Pre-existing medical conditions				
Mental disorder	<0.001*	0.002*	0.019	0.288
Other cardiovascular	0.457	0.907	0.006*	0.116
Asthma	0.633	0.875	0.626	0.931
Diabetes mellitus type 2	0.297	0.534	0.089	0.648
Allergy	0.259	0.738	0.124	0.609
Thyroid illness	0.884	0.493	0.542	0.454
Other autoimmune	0.244	0.028	0.117	0.085
Hypertension	0.636	0.59	0.602	0.675
Diabetes mellitus type 1	0.849	0.938	0.588	0.896

*Indicates a significant p-value after correction for a false discovery rate of 10%.

P-values are from univariable negative binomial regression models (WHODAS 2.0) and ordinal regression models (self-rated health).

Table S8. Association between time since positive test, WHODAS 2.0 score and self-rated health in subacute and postcovid phase participants

	Univariable			Multivariable		
**Self-rated health**	OR	95% CI	P	OR	95% CI	P
Postcovid phase	0.98	0.94-1.01	0.130	0.98	0.95-1.02	0.293
Subacute phase	0.75	0.63-0.89	0.001	0.71	0.60-0.85	<0.001
**WHODAS 2.0**	MR	95% CI	P	MR	95% CI	P
Postcovid phase	1.00	0.98-1.03	0.859	0.99	0.97-1.02	0.642
Subacute phase	0.69	0.61-0.78	<0.001	0.66	0.58-0.74	<0.001
OR: Odds ratio per four-week period for a worse rating of self-rated health, estimated with ordinal logistic regression.						
MR: mean ratio per four-week period of WHODAS 2.0 sum score, estimated with negative binomial regression.						
An OR<1 or MR<1 represents better self-rated health or lower WHODAS 2.0 score with longer time since positive PCR.						
Multivariable model: adjusted for illness severity at time of testing, occupation group and COVID-19 exposure at work						

STROBE Statement—checklist of items that should be included in reports of observational studies
Larsson et al., Self-reported symptom severity, general health, and impairment in post-acute phases of COVID-19: retrospective cohort study of Swedish public employees

	Item No.	Recommendation	Page No.	Relevant text from manuscript
**Title and abstract**	1	(*a*) Indicate the study's design with a commonly used term in the title or the abstract	1-2	"retrospective cohort study of Swedish public employees"
		(*b*) Provide in the abstract an informative and balanced summary of what was done and what was found	2	Abstract
**Introduction**				
Background/rationale	2	Explain the scientific background and rationale for the investigation being reported	3	"these symptoms are among the most frequently occurring symptoms in the general population. Therefore, in order to establish valid diagnostic criteria, and to estimate contribution to disease and impairment, it is important to examine how the health of persons in the post-acute phase of COVID-19 differ from comparable uninfected individuals"
Objectives	3	State specific objectives, including any prespecified hypotheses	4	"to examine the long-term excess in symptom severity and functional impairment following COVID-19, we designed a study…"
**Methods**				
Study design	4	Present key elements of study design early in the paper	4	"...public employees included in an extensive PCR testing program, irrespective of SARS-CoV-2 positivity, were invited to complete an online survey…"
Setting	5	Describe the setting, locations, and relevant dates, including periods of recruitment, exposure, follow-up, and data collection	4, 11	"Starting in March, 2020, Region Västra Götaland … offered their employees

				PCR testing for presence of SARS-CoV-2 in case of symptoms suggestive of COVID-19.”
Participants	6	(*a*) *Cohort study*—Give the eligibility criteria, and the sources and methods of selection of participants. Describe methods of follow-up<br>*Case-control study*—Give the eligibility criteria, and the sources and methods of case ascertainment and control selection. Give the rationale for the choice of cases and controls<br>*Cross-sectional study*—Give the eligibility criteria, and the sources and methods of selection of participants	6	“As the present study focused on long-term general health and symptoms of those with PCR verified SARS-CoV-2 infection in relation to comparable controls, we applied the following exclusion criteria:…”
		(*b*) *Cohort study*—For matched studies, give matching criteria and number of exposed and unexposed<br>*Case-control study*—For matched studies, give matching criteria and the number of controls per case		
Variables	7	Clearly define all outcomes, exposures, predictors, potential confounders, and effect modifiers. Give diagnostic criteria, if applicable	6-10	
Data sources/ measurement	8*	For each variable of interest, give sources of data and details of methods of assessment (measurement). Describe comparability of assessment methods if there is more than one group	6-10	
Bias	9	Describe any efforts to address potential sources of bias	6	“Inclusion criteria…: (v) negative test within 4 weeks of completing the survey (to minimize the possibility that current health and symptoms was affected by other acute illness).”
Study size	10	Explain how the study size was arrived at	5, 11, Figure 1	“From a database at the Sahlgrenska University Hospital, we identified 56,483 individuals >18

years old who had registered their Swedish personal identification number, mobile phone number and home address to receive at least one employer-ordered test for presence of SARS-CoV-2 by PCR between March 26th and November 30th, 2020.”

Continued on next page

Quantitative variables	11	Explain how quantitative variables were handled in the analyses. If applicable, describe which groupings were chosen and why	6	"We divided our sample into three groups: those in the subacute phase after testing positive (>4 - ≤12 weeks from the survey, hereafter referred to as 'subacute') and those in the postcovid phase after testing positive (>12 weeks from the survey, hereafter referred to as 'postcovid') and those who tested negative for SARS-CoV-2 with PCR (hereafter referred to as 'PCR negative'). " … "Our main interest was to examine general health and symptom

				severity in postcovid participants, compared to PCR-negative participants. To examine the dynamics of symptoms and general health following COVID-19 we also wanted to compare postcovid participants to subacute participants.”
Statistical methods	12	(*a*) Describe all statistical methods, including those used to control for confounding	9-10	
		(*b*) Describe any methods used to examine subgroups and interactions	9	“Next, we explored if determinants of WHODAS 2.0 score and self-rated health differed depending on COVID-19 status. We conducted models including each variable of interest, COVID-19 status and their interaction term.”
		(*c*) Explain how missing data were addressed	5	“We could only view and export data from participants who had completed and submitted the questionnaire in its whole.”… “BMI could

				not be calculated for five participants due to obviously unlikely reported values.”
		(*d*) *Cohort study*—If applicable, explain how loss to follow-up was addressed<br>*Case-control study*—If applicable, explain how matching of cases and controls was addressed<br>*Cross-sectional study*—If applicable, describe analytical methods taking account of sampling strategy	9	“We adjusted [our analyses]} for exposure to COVID-19 patients at work and occupation, since these two variables could act as confounders.”
		(*e*) Describe any sensitivity analyses	-	None performed
**Results**				
Participants	13*	(a) Report numbers of individuals at each stage of study—eg numbers potentially eligible, examined for eligibility, confirmed eligible, included in the study, completing follow-up, and analysed	11, Figure 1	
		(b) Give reasons for non-participation at each stage	Figure 1	
		(c) Consider use of a flow diagram	Figure 1	
Descriptive data	14*	(a) Give characteristics of study participants (eg demographic, clinical, social) and information on exposures and potential confounders	11-12, Table 1	“Table 1 presents characteristics of the subacute, postcovid and PCR negative groups. The median age was 45 years and about 85% were women.”
		(b) Indicate number of participants with missing data for each variable of interest	Table 1	
		(c) *Cohort study*—Summarise follow-up time (eg, average and total amount)	-	
Outcome data	15*	*Cohort study*—Report numbers of outcome events or summary measures over time	11	“Weeks (IQR) between test and survey were a median of 8.4 (6.4-10.1) for subacute, 31.8 (14.4-37.4) for postcovid and 23.0 (15.7-33.7) for PCR negative participants.”
		Case-control study—Report numbers in each exposure category, or summary measures of exposure	-	

		Cross-sectional study—Report numbers of outcome events or summary measures	-
Main results	16	(*a*) Give unadjusted estimates and, if applicable, confounder-adjusted estimates and their precision (eg, 95% confidence interval). Make clear which confounders were adjusted for and why they were included	Figures and Supplementary tables
		(*b*) Report category boundaries when continuous variables were categorized	Figure 4, Table S5-S6
		(*c*) If relevant, consider translating estimates of relative risk into absolute risk for a meaningful time period	-

Continued on next page

Other analyses	17	Report other analyses done—eg analyses of subgroups and interactions, and sensitivity analyses	13, Figure 4	"To examine if the association between COVID-19 status and WHODAS 2.0 or self-rated health was dependent on other factors, we conducted interaction analyses."
**Discussion**				
Key results	18	Summarise key results with reference to study objectives	14	"We found that having had COVID-19, verified by PCR, was associated with poorer self-rated health and functioning, and more severe symptoms when compared with patients without a history of COVID-19. These associations were clearly strongest in the subacute phase, but persisted to a lesser extent into the postcovid phase."
Limitations	19	Discuss limitations of the study, taking into account sources of potential bias or imprecision. Discuss both direction and magnitude of any potential bias	17-18	"Strengths of the current study include the relatively large number of participants, the comprehensive survey, objectively verified infection

				and the presence of negative controls. Our study also has several limitations… ”
Interpretation	20	Give a cautious overall interpretation of results considering objectives, limitations, multiplicity of analyses, results from similar studies, and other relevant evidence	14-19	Discussion section
Generalisability	21	Discuss the generalisability (external validity) of the study results	18	“Secondly, several factors limit the generalizability of our study.”
**Other information**				
Funding	22	Give the source of funding and the role of the funders for the present study and, if applicable, for the original study on which the present article is based	23-24	“

*Give information separately for cases and controls in case-control studies and, if applicable, for exposed and unexposed groups in cohort and cross-sectional studies.

Note: An Explanation and Elaboration article discusses each checklist item and gives methodological background and published examples of transparent reporting. The STROBE checklist is best used in conjunction with this article (freely available on the Web sites of PLoS Medicine at http://www.plosmedicine.org/, Annals of Internal Medicine at http://www.annals.org/, and Epidemiology at http://www.epidem.com/). Information on the STROBE Initiative is available at www.strobe-statement.org.
